# Medical Oxygen as a Life-Saving Medicine: A rapid review of the oxygen landscape and innovative efforts in the World Health Organization Eastern Mediterranean Region in Response to COVID-19 and Beyond

**DOI:** 10.18103/mra.v11i7.2.4162

**Published:** 2023-07-29

**Authors:** Chiori Kodama, Gary Kuniyoshi, Shaffi Koya, Mohamed Salem, Mostafa Monier Othman, Kazuyo Iwamoto, Abdinasir Abubakar, Richard Brennan

**Affiliations:** 1 WHO Health Emergencies Programme, World Health Organization Eastern Mediterranean Regional Office, Cairo, Egypt

**Keywords:** Medical Oxygen, COVID-19, mortality, WHO Eastern Mediterranean Region, Workforce, Live oxygen platform, Oxygen scale-up, Oxygen roadmap

## Abstract

**Background:**

Medical oxygen is an essential treatment for life-threatening hypoxemic conditions and is commonly indicated for the clinical management of many leading causes of mortality. Many countries of the World Health Organization (WHO) Eastern Mediterranean Region (EMR) lacked robust medical oxygen systems prior to the COVID-19 (corona virus disease) pandemic and this situation was exacerbated by increased needs, particularly in remote and rural health facilities, resulting in many unfortunate deaths. The aim of this article is to describe the oxygen landscape in the region and the regional initiatives undertaken by countries and WHO.

**Methodology:**

We conducted a rapid review to synthesize the available literature on the needs and availability of oxygen and its related resources and the regional initiatives undertaken. We conducted search in PubMed, relevant WHO and World Bank websites, and in general using google to understand the health of conditions that could benefit from the availability of medical oxygen, oxygen related resources including health workforce available for support and usage of medical oxygen, and the initiatives by WHO, countries and partners to improve the situation. We used a snowballing technique and reviewed all available databases for reports, surveys, assessments, and studies related to medical oxygen, besides WHO internal records, assessments, and consultation reports.

**Results:**

The data on oxygen availability, supply demand gap, infrastructure facilities, and human resources were sparse. The regional initiatives have led to increase in resources, including human resources and oxygen production infrastructure. *The Live Oxygen Platform* (LOP), contributed to improved availability of quality data needed for supply demand assessments.

**Conclusion:**

A regional enterprise strategy to promote sustainable, decentralized, and contextualized production, supply, and monitoring of oxygen together with human resource support including training and placement by WHO, partners, and governments contributed to improved availability of oxygen in the region. Additionally, with the LOP, governments, WHO, and partners have access to better data availability for policy decision making and timely resource allocation.

## Introduction

Medical oxygen is essential for the treatment of hypoxaemia across many communicable and noncommunicable diseases and medical conditions across the life course, to which older persons and children, in particular, are vulnerable. In 2017, oxygen was added to the World Health Organization (WHO) Model List of Essential Medicines for treating hypoxaemia, (1) with its utility spanning a diverse range of clinical disciplines including paediatrics, neonatology, obstetrics, surgery, and trauma. These include diseases like cardiovascular diseases, pneumonia, tuberculosis, and chronic obstructive pulmonary disease, medical conditions requiring surgery, and emergency and critical care. Coronavirus disease (COVID-19) exacerbated the need for oxygen in pandemic situations, demonstrated the role of oxygen in better outcomes, and exposed the lack of medical oxygen as a critical gap in the healthcare systems in many countries. (2 – 5)

Countries in the WHO Eastern Mediterranean Region (EMR) vary greatly in resources, growth indices, and economic strengths, with more than 102 million requiring humanitarian assistance, (6) many of which result from armed conflicts, political instability or insecurity, or natural disasters. These emergencies have significantly weakened or disrupted health systems. The region is home to 43% of those who need humanitarian assistance, the source of 64% of the world’s refugees (7) and nearly one-third of the world’s internally displaced persons. (8) These populations are vulnerable due to poor living conditions, and many remain marginalized with limited access to needed quality health care, including medical oxygen.

However, in the EMR, access to medical oxygen has been interrupted at many facilities. That lack of access is contributing to preventable deaths. This problem has been aggravated by the COVID-19 pandemic when the need for medical oxygen has exceeded the capacities of many health systems. Medical oxygen is often overlooked in health system planning because of complex infrastructure requirements, perceptions about cost, and poor understanding of its health impact. Almost half of all hospitals in low and middle-income countries have an inconsistent supply of medical oxygen or lack it entirely. (9) In resource-constrained settings, access to medical oxygen is unreliable due to cost, distance from production centres, undermaintained infrastructure, and a fragmented supply chain.

It was in this context that the first part of this study was conducted to rapidly review and understand the situation of oxygen supply and demand in the region. Later, the study was expanded to have a rapid review to synthesize the initiatives by WHO, Member States (MS), and partners to improve the oxygen infrastructure and availability.

## Methods

We followed a rapid review method (10) that international agencies and governments have long used in making policy and programme decisions, especially in emergencies. (10, 11). We had three objectives in undertaking this review— one, to understand the burden of diseases in the countries of the region that will benefit from better oxygen availability, two, to understand the availability of oxygen and related resources in the region, and three, to describe the initiatives undertaken by WHO, MS, and partners to improve the situation.

For the first two objectives, we tailored the literature search, starting with a search in the PubMed database for peer reviewed publications. We used a generic search string combining terms for medical oxygen, and the names of all MS names (Supplement S1 for the complete string). We included only English language studies published in the most recent five years (2017–2021) to get the most recent data. Only one researcher conducted the search, study selection, and data abstraction, with verification by a second reviewer, as is common for rapid reviews (12). Additionally, we reviewed WHO regional and country websites, relevant WHO and World Bank documents and reports, including unpublished reports, and finally, using a snowballing approach, identified other databases to understand the oxygen landscape. We conducted the landscape review in January 2021 and updated the results in December 2021. For the third objective of reviewing the initiates, we largely screened WHO documents and reports and the *Live Oxygen Platform* (LOP) hosted and maintained by WHO, where MS share data regarding hospitals and oxygen facilities. This second part of the review was conducted in March 2023. We used a descriptive synthesis method to summarize our findings from both the sub-reviews.

## Results

### The oxygen resources landscape analysis for the region

The PubMed search resulted in 711 papers, of which there were 24 review papers. None of these reviews, including the 10 systematic reviews, discussed the medical oxygen availability in the region or MS. The only paper that discussed oxygen supply, demand, or related infrastructure was by Amira K Al-Aamri et al., which forecasted the critical care resources threshold in the Gulf Cooperation Council (GCC) countries. (13) The paper predicted the number of critical cases requiring oxygen, among many other parameters estimated using the WHO Adaptt Surge Planning Tool. (14). The study predicted that the maximum number of COVID-19 patients needing oxygen therapy during the peak of emergency admissions varies between 690 in Bahrain, 1,440 in Oman, and over 10,000 in Saudi Arabia.

With the sparse availability of data in peer reviewed literature, we turned to other databases to understand the oxygen production and consumption in the Region. We used the WHO mortality database to determine the number of deaths due to diseases and conditions requiring oxygen therapy in the region. (Table 1)

A significant number of deaths related to perinatal conditions, respiratory conditions, and injuries may benefit from a more organized and sustainable oxygen delivery in the region. Our review also showed a serious gap in our understanding of the oxygen requirements in the region, for example, we needed data for Afghanistan, Djibouti, Libya, Morocco, Pakistan, Palestine, Saudi Arabia, Somalia, Sudan, Tunisia, and Yemen.

One of the key determinants of actual oxygen demand irrespective of the medical conditions requiring oxygen is the availability of medical facilities where oxygen can be administered, including the availability of hospital beds, Intensive Care Unit (ICU) beds, and mechanical ventilators. Understanding these determinants is important in calculating oxygen needs. We used the World Bank database to calculate the hospital and bed capacities that largely determine national medical oxygen consumption in the region. (Table 2).

One of the most essential requirements to have a sustainable system for oxygen production, maintenance of facilities, and supply chain is the availability of key human resources. However, we found very little data on the number of Biomedical Engineers and Technicians in MS. (Table 3)

The number of biomedical engineers ranged from single digits in countries like Afghanistan and Djibouti to 1000 or more in Egypt, Jordan, and Pakistan. We also found wide variations in the definitions used for the critical human resources reported, including ICU physicians and nurses among the countries. The number of Physicians per 1,000 population, which also critically affect healthcare delivery, and determines the use of oxygen facilities, ranged from less than 0.1 in Somalia, 0.2 in Djibouti, 0.3 in Afghanistan and Sudan to 2.7 in Jordan and Saudi Arabia.

### Regional initiatives

#### Development of the Live Oxygen Platform (LOP)

Our review of WHO consultation documents and reports showed the need for a uniform database or repository that collectively captures oxygen production and consumption data at national and sub-national levels. However, we identified some international databases and platforms which perform similar but only partial functions (See Supplement Table S3 to see the list of platforms reviewed). We then examined the WHO initiative called *Live Oxygen Platform* (LOP) (20). This is an online data platform on oxygen capacity and needs in MS for timely public response and was a direct result of the WHO technical consultation on oxygen access scale-up for COVID-19, which was convened in October-November 2020. (21) This platform was designed to serve as a centralized repository of information and tools and operates as a ‘global good’, a platform that carries out real time aggregation, analysis, and interpretation of medical oxygen access scale-up activities in different contexts, with information captured in both prospective and retrospective manners. The development of the actual platform started with determining the “metadata” required to develop clear and targeted objectives for the platform. This came from an in-depth exploration of existing data platforms to see if any could be adapted or modified specifically for medical oxygen and have broader and more in-depth data capture. (22–27) However, the review could not find a single repository that serves as a live tool for mapping of oxygen supply and its allocation and gaps across a country and administrative levels that give insight into the efficiency of the entire oxygen ecosystem.

##### National biomedical engineers

Our review also identified how the WHO and MS responded to the critical need for biomedical engineer support by supporting resource limited and conflict affected countries with national biomedical engineers (NBME). Our review of the terms of references and advertisements for the positions showed that preference was given to nationals since they better understand the context of the oxygen situation and relevant background of the countries and have access to hard-to-reach areas where foreign experts are not permitted to enter. NBMEs who could support on assessing overall biomedical demands and providing training and technical advice to health authorities and healthcare workers in oxygen and biomedical equipment use at the facility level were given preference.

Documents reviewed showed that 17 NBMEs have been successfully identified and assigned, playing a vital role in the 10 most resource limited and complex emergency countries for scaling up of oxygen with the support of UNITAID— a global health initiative mainly funded by some countries and Foundations. (28) The number of NBMEs assigned are as follows (updated as of June 10, 2023): Afghanistan (2), Djibouti (1), Iraq (1), Lebanon (2), Libya (1), Pakistan (3), Somalia (3), Sudan (1), Syria (1) and Yemen (2). The contribution of NBMEs in scaling up of national oxygen capacities has been indispensable and well appreciated by the countries, as per minutes of meetings and internal WHO reports. The list of duties performed by the NBMEs are listed in Supplement Table S4.

#### Development of a regional oxygen scale-up roadmap

We reviewed the process in which the draft regional roadmap for oxygen scale-up was developed. Our review showed that the roadmap was built on the experience gained in the use of medical oxygen during the COVID-19 pandemic and previous emergencies and that six documents considered international level guidance on oxygen scale-up were studied in the preparation of the regional roadmap. These include a technical consultation report, (21) recommendations on designing country roadmaps, (29, 30) systematic approaches, (31, 32) and principal examples of projects in countries. (33, 34) The road map also used national oxygen roadmaps or scale-up implementation plans from five countries, namely Ethiopia, (35) Liberia, (36) Malawi, (37) Nigeria, (38) and Uganda. (39) These country plans were supported by one or more partners such as Clinton Health Access Initiative (CHAI), Program for Appropriate Technology in Health (PATH), United Nations Children’s Fund (UNICEF), or United States Agency for International Development (USAID). The Ethiopian national medical oxygen and pulse oximetry scale-up road map (34) which the Ethiopian Ministry of Health (MOH) designed and implemented with support from partners in 2016, was particularly referred to. The WHO EMR regional roadmap for oxygen scale-up was developed and finalized in 2022 based on all these inputs.

## Discussion

To our understanding, this is the first published review of the region’s oxygen supply and demand landscape and the regional initiatives to overcome the challenges. This review comes at the backdrop of the seventy-sixth World Health Assembly (WHA) resolution in May 2023 (40), which calls for increasing access to medical oxygen globally. Through 20 recommendations, the resolution suggests that MS consider their national contexts when planning oxygen scale-up.

Some of the key findings related to medical oxygen through the landscape analysis are as follows: limited availability of data on companies producing medical oxygen in the region, very few countries in the region have national policies relating to medical oxygen, use assessment tools for medical oxygen at the facility level, and have updated national treatment guidelines for conditions that may require oxygen therapy such as sepsis, pneumonia, and chronic obstructive pulmonary disease. No country in the region has had a WHO technical consultation on oxygen access scale-up or a published national medical oxygen scale-up roadmap, implementation plan, or best practices document. Similarly, the review of the available national documents demonstrated a lack of guidance to support oxygen delivery systems and clinical management across EMR and that many facilities may be ill-equipped to develop new oxygen capacities, provide oxygen to those who need it, and titrate or discontinue oxygen appropriately.

In response, WHO developed a series of initiatives, including trainings, positioning of NBMEs, and developing LOP following consultative meetings and the landscape analysis for the Region. The LOP was officially launched in November 2021, and this was followed by data collection on hospitals and companies producing oxygen. During the initial implementation of the LOP, important opportunities and challenges were discovered. The platform had its challenge regarding data security and sensitivity. One example was the difficulty in mapping the actual locations of facilities (hospitals or companies) using Global Positioning System (GPS) coordinates. It was determined early on not to map specific locations of hospitals or companies for security reasons, particularly in conflict-affected settings. The platform, designed to be a networking tool in addition to a dashboard, is still in evolving and is currently being used by a restricted number of national users with access to the platform. The data are currently collected by the ministries of health directly or through local focal points, WHO country office staff, or NBMEs during field visits. As of March 2023, 16 countries out of 22 member states in the region are participating in this innovative oxygen data platform. In the next phase, the network of the health care facilities providing care to patients or companies producing oxygen or biomedical supplies will have direct access to enter their data. This can be achieved by extensive stakeholder engagement beyond mere consultation and confidence building and requires concerted effort by national, regional, and local governments, ministries, policy experts, health care workers, and health facility leadership.

The regional oxygen roadmap recommends some key activities to achieve oxygen scale up. These include one, national medical oxygen needs assessment in a holistic and comprehensive manner, two, national technical guidance, operational tools and quality standards for production and use of medical oxygen, and three, national oxygen scale-up dashboard for mapping medical oxygen and as a networking resource. First, oxygen needs assessments must be performed at the national and subnational level and results made readily available and accessible to health care facilities providing care to patients. Stakeholder engagement is key to this process and needs assessments should be done to ensure facilities and communities are involved in decision-making. Second, national guidance’s need to be available on appropriate procurement, maintenance, and education, to scale-up and use oxygen appropriately and effectively. Guidance for facilities developing new oxygen generation capabilities is needed. Identifying patients who need oxygen therapy and appropriately initiating, titrating, and discontinuing oxygen therapy is likewise essential. These national guidance’s should be readily available and easy-to-use. Education and training of health care workers and biomedical professionals can ensure that resources are being used efficiently. Third, monitoring the production and consumption of medical oxygen should be done at the national and subnational level.

The regional innovations through COVID-19 response have provided support to the sustainable provision of medical oxygen in the countries. For example, the solar-powered oxygen system in Somalia has already shown results where pneumonia accounts for at least one-fifth (N=15,160) of deaths among children under five years. (41–43) All of the countries classified as FCV (Fragile, Conflict-affected, and Vulnerable) settings in the region are actively participating in the LOP. The WHO recruited NBMEs for resource limited countries have started filling the local gaps where the needs and gaps varied widely across and within countries— from lack of national guidance and regulation in oxygen scale-up, to maintenance and repair of PSA plants or biomedical equipment and safe use of medical oxygen, and to various types of biomedical trainings to clinicians, technicians, and hospital managers etc.

Our review identified a few challenges amidst all the initiatives, and these include one, poor involvement of external partners in the region due to the complexities and insecurity in conflict-affected countries, two, data sensitivities and reluctance to sharing data leading to a few countries withdrawing from sharing data with LOP at some point, three, lack of local human health resources like biomedical engineers and technicians and the lack of training facilities, and four, delays in the data collection process for LOP.

## Conclusion

Diseases requiring oxygen therapy remain a leading cause of death and disability in the region. Although efforts are being made to address prevention of these diseases, considerable strides are needed to reduce the burden of these diseases and resulting mortality among infants, children, and adults including pregnant womene. The review showed not only the poor availability of oxygen production facilities, and human resources, but also the challenges regarding data availability. The regional roadmap for oxygen scale-up and novel endeavours including the LOP are expected to support the countries in the long term. The regional enterprise strategy to promote sustainable and decentralized production and supply of oxygen requires commitments and prioritization of several activities. These include securing the contracts of NBME for long term, development of country roadmaps and long-term strategy to achieve sustainability, securing funding and infrastructure, involvement of partners to complement various areas of oxygen scale-up, among others.

## Supplementary Material

1

## Figures and Tables

**Table 1.** Death rates of conditions that could benefit from the availability of medical oxygen^#^

Country	No. of death by CVDs	No of death by Perinatal conditions	No of death by respiratory infections	No of death by injuries
Bahrain	865 (2014)	77 (2014)	29 (2014)	210 (2014)
Egypt	306,363 (2019)	3,055 (2019)	19,609 (2019)	16,670 (2019)
Iran	141,693 (2016)	11,181 (2016)	8,243 (2016)	32,880 (2016)
Iraq	44,202 (2016)	7,742 (2016)	2,085 (2016)	14,578 (2016)
Jordan	7,064 (2018)	104 (2018)	461 (2018)	1,024 (2018)
Kuwait	2,996 (2019)	201 (2019)	114 (2019)	864 (2019)
Lebanon	4,110 (2021)	966 (2021)	1,308 (2021)	986 (2021)
Oman	1,076 (2021)	100 (2021)	2,998 (2021)	350 (2021)
Qatar	798 (2021)	74 (2021)	170 (2021)	373 (2021)
Syria	33,780 (2010)	132 (2010)	2,008 (2010)	3,088 (2010)
UAE	2,078 (2020)	170 (2020)	1,107 (2020)	900 (2020)

# WHO platform (https://platform.who.int/mortality/themes/theme-details/MDB/all-causes); (15) CVDs-cardiovascular diseases

**Table 2.** Hospital and bed capacities that largely determine national medical oxygen consumption^#^

Country	Number of Hospitals	Hospital beds per 1,000 population* (2017)	Intensive Care Unit (ICU) beds per 100,000 population** (2020)	Mechanical ventilators^
Afghanistan	150	0.4	1.63 (0.8)	300
Bahrain	29	1.7	3.57 (7.1)	
Djibouti	16	1.4	2.38 (3.3)	
Egypt	1,300	1.4	2.38 (3.8)	
Iran	954	1.6	3.32 (5.0)	
Iraq	372	1.3	3.32 (4.6)	
Jordan	117	1.5	3.32 (4.6)	
Kuwait	44	2.0	3.57 (7.1)	
Lebanon	130	2.7	3.32 (9.6)	1250
Libya	96	3.2	3.32 (12)	350
Morocco	534	1.0	2.38 (2.6)	
Oman	69	1.5	3.57 (5.7)	
Pakistan	1979	0.6	2.38 (1.4)	2200
Qatar	16	1.3	3.57 (4.3)	
Saudi Arabia	540	2.2	3.57 (9.7)	
Somalia	77	0.7	1.63 (1.5)	
Sudan	272	0.8	2.38 (2.4)	84
Syria	91	1.4	1.63 (2.4)	153^$^
Tunisia	184	2.2	2.38 (5.5)	
UAE	134	1.4	3.57 (4.3)	
Palestine	35	1.2	2.38 (2.9)	342
Yemen	275	0.7	1.63 (1.1)	500

# World Bank (WB, 2023). (16) Note: Empty cells indicate that no data were available. The data of non-invasive devices is missing;

** Calculation from population (UNDP), total hospital bed (WB) and %ICU bed (WB) from consolidates all HCW, lab and bed estimates from 2020, found in WHO Essential Supplies Forecasting tool.

$ NWS: Northwest Syria.

^ The sources for the mechanical ventilator data are included in Supplement. (S2)

**Table 3.** Human resources available for support and usage of medical oxygen

Country	Biomedical Engineers (2017)	Biomedical Technicians (2017)	Nurses^@^ per 1,000 people	Physicians per 1,000 people
Afghanistan	>1		0.4 (2018)	0.3 (2020)
Bahrain	2		2.5 (2015)	0.9 (2020)
Djibouti	>1		0.7 (2014)	0.2 (2014)
Egypt	1000		1.9 (2018)	0.7 (2017)
Iran	>1		2.1 (2018)	1.6 (2018)
Iraq			2.4 (2020)	1.0 (2020)
Jordan	2370	1140	3.3 (2019)	2.7 (2020)
Kuwait	>1		7.4 (2018)	2.3 (2020)
Lebanon	750		1.7 (2018)	2.2 (2019)
Libya	40	40	6.5 (2017)	2.1 (2017)
Morocco			1.4 (2017)	0.7 (2017)
Oman			3.9 (2020)	1.8 (2020)
Pakistan	1200		0.5 (2019)	1.1 (2019)
Qatar			7.2 (2018)	2.5 (2018)
Saudi Arabia	300		5.8 (2019)	2.7 (2020)
Somalia			0.1 (2014)	0.0 (2014)
Sudan	365		1.1 (2018)	0.3 (2017)
Syria			1.5 (2016)	1.3 (2016)
Tunisia	20		2.5 (2017)	1.3 (2017)
UAE	3		5.7 (2019)	2.6 (2019)
Palestine			no data	0.8 (2001)
Yemen	150	300	0.8 (2018)	0.5 (2014)

# WHO Global Health Observatory;

$ World Bank;

@ Nurses and midwives (17–19)

## Data Availability

Some of the data that supports the findings of the current study are not publicly available due to data protection policies. Data are however available from the corresponding author on reasonable request. Other data are available from public sources like World Bank and WHO websites.
