# Supplementary material for: Medical Oxygen as a Life-Saving Medicine: A rapid review of the oxygen landscape and innovative efforts in the World Health Organization Eastern Mediterranean Region in Response to COVID-19 and Beyond
Source: Med Res Arch. Author manuscript; Available in PMC 2023 Aug 21. (PMC10440734; doi:10.18103/mra.v11i7.2.4162)
Supplement: 1 [file NIHMS1923388-supplement-1.pdf]

SUPPLEMENT FILES

Supplement S1: Search string used in PubMed.

(("oxygen"[Text Word] OR "medical oxygen"[Text Word]) AND ("Afghanistan"[Text Word] OR "Bahrain"[Text Word] OR "Djibouti"[Text Word] OR "Egypt"[Text Word] OR "Iran"[Text Word] OR "Iraq"[Text Word] OR "Jordan"[Text Word] OR "Kuwait"[Text Word] OR "Lebanon"[Text Word] OR "Libya"[Text Word] OR "Morocco"[Text Word] OR "Oman"[Text Word] OR "Pakistan"[Text Word] OR "Qatar"[Text Word] OR "Saudi Arabia"[Text Word] OR "Somalia"[Text Word] OR "Sudan"[Text Word] OR "Syria"[Text Word] OR "Tunisia"[Text Word] OR "UAE"[Text Word] OR "Palestine"[Text Word] OR "Yemen"[Text Word])) AND (2017:2021[pdat])

Supplement Table S2: Data sources for mechanical ventilator availability

Afghanistan: https://tolonews.com/afghanistan/only-300-ventilators-afghanistan-treat-C-19-moph
Libya: https://www.nytimes.com/2020/04/18/world/africa/africa-coronavirus-ventilators.html
Lebanon: https://sites.aub.edu.lb/maingate/2020/08/07/ventilators-made-to-order/
Pakistan: https://www.dawn.com/news/1543410
Sudan: https://www.nytimes.com/2020/04/18/world/africa/africa-coronavirus-ventilators.html
Syria: https://reliefweb.int/report/yemen/more-15-million-children-and-their-families-yemen-syria-and-gaza-set-face-C-19
Palestine: https://www.timesofisrael.com/80-90-of-ventilators-in-west-bank-and-gaza-already-in-use-who-official-says/
Yemen: https://reliefweb.int/report/yemen/more-15-million-children-and-their-families-yemen-syria-and-gaza-set-face-C-19

Table S3: List of reviewed existing visual trackers or mapping platforms of medical oxygen or biomedical equipment

Developer	Title of tool	Purpose	Limitations
Gasworld	Oxygen mapping production network	Country oxygen industrial producers	Only industrial plants mapped
National Institute of Industrial Engineering (India)	Dynamic allocation of liquid oxygen dashboard	India oxygen supply and demand by region	Only liquid oxygen supply and demand
UNICEF	Oxygen System Planning Tool	Country oxygen availability	Only oxygen availability
PATH	C-19 Oxygen needs tracker	Country daily oxygen need for C-19, (data used to calculate the daily oxygen need across LMICs is pulled from the World Health Organization (WHO) Coronavirus Disease (C-19) Dashboard.)	Only oxygen daily need
PATH	Sub-Saharan Africa Respiratory Care Equipment Distributors	Country respiratory care supplier	Only suppliers mapped

Table S4: List of duties as per Terms of Reference for NBMEs

1.Conduct the oxygen data collection from facilities and suppliers, ensure to capture the baseline oxygen production capacity and oxygen supply system in assigned country<br>2.Based on the assessment in the oxygen mapping, support estimation of oxygen demand and gap in assigned country<br>3.Based on the assessment in the oxygen mapping, support development of a fit-for-purpose oxygen scale up plan for assigned country including technical support to the national, subnational or health facility level to plan their oxygen supply system, from the oxygen source to the patient delivery device<br>4.Based on the assessment in the oxygen mapping, support development of bundles to ensure long term operations of the equipment, training gaps, Infection Prevention and Control measure gaps, and maintenance gaps. Plan a strategy and cost<br>5.Train healthcare workers in oxygen and biomedical equipment use<br>6.Identification of the medical equipment and necessary additional components requirements as requested by respective local ministry/partners and define and advise WHO on the installation constraints of equipment related to works<br>7.Equipment installation/repair/maintenance completion reports and certification when required.

NBME: National Biomedical Engineers
